# Regulation of apoptosis and priming of neutrophil oxidative burst by diisopropyl fluorophosphate

**DOI:** 10.1186/1476-9255-7-32

**Published:** 2010-07-07

**Authors:** Jennifer LY Tsang, Jean C Parodo, John C Marshall

**Affiliations:** 1 Interdepartmental Division of Critical Care, University of Toronto, Toronto, Canada; 2 Departments of Critical Care Medicine and Surgery, Saint Michael's Hospital, Room 4-007, Bond Wing, 30 Bond Street, Toronto, Ontario, M5B 1W8, Canada; 3 Department of Critical Care Medicine, Sunnybrook Health Sciences Centre, 2075 Bayview Avenue, Room D112, Toronto, Ontario, M4N 3M5, Canada

## Abstract

**Background:**

Diisopropyl fluorophosphate (DFP) is a serine protease inhibitor that is widely used as an inhibitor of endogenous proteases in *in vitro *neutrophil studies. Its effects on neutrophil function are unclear. We sought to determine the biological effects of DFP on human neutrophil apoptosis and oxidative burst.

**Methods:**

We isolated neutrophils from healthy volunteers, incubated them with DFP (2.5 mM), and evaluated neutrophil elastase (NE) activity, neutrophil degranulation, apoptosis as reflected in hypodiploid DNA formation and exteriorization of phosphatidylserine (PS), processing and activity of caspases-3 and -8, oxidative burst activity and hydrogen peroxide release.

**Results:**

Consistent with its activity as a serine protease inhibitor, DFP significantly inhibited NE activity but not the degranulation of azurophilic granules. DFP inhibited constitutive neutrophil apoptosis as reflected in DNA fragmentation, and the processing and activity of caspases-3 and -8. DFP also inhibited priming of neutrophils for oxidative burst activity and hydrogen peroxide release. However, DFP enhanced the exteriorization of PS in a dose-dependent manner.

**Conclusion:**

We conclude that DFP exerts significant effects on neutrophil inflammatory function that may confound the interpretation of studies that use it for its antiprotease activity. We further conclude that endogenous proteases play a role in the biology of constitutive neutrophil apoptosis.

## Background

Diisopropyl fluorophosphate (DFP) is an irreversible serine protease inhibitor. Its hydrophobic nature and low molecular weight allow it to permeate intact cells and intracellular granules to prevent proteolysis before cellular barriers are disrupted by homogenization or detergent [1]. Since neutrophil granules contain potent endogenous proteases, DFP is commonly used in neutrophil studies to prevent degradation of proteins [1]. In addition to its use as a protease inhibitor, the radioactive form of DFP has been used to label granulocytes to study neutrophil kinetics in humans [2].

Neutrophil serine proteases - cathepsin G, neutrophil elastase (NE) and proteinase 3 are enzymes that are stored in azurophilic granules and are important in intracellular microbial killing [3]. NE has many physiological roles, including the regulation of neutrophil chemotaxis [4], adhesion [5] and migration [6]. However, excessive NE can result in cell and tissue injury by compromising the integrity of endothelial vascular barrier and promoting microvascular injury, resulting in increased permeability and interstitial edema [7,8]. NE can also induce the expression and release of IL-8 - a potent neutrophil chemoattractant that promotes neutrophil recruitment [9,10], release of granular enzymes and respiratory burst activity [11].

Studies of the biological effects of DFP on cell types other than neutrophils are abundant. For example, it has been previously shown that DFP can block T cell receptor-triggered apoptosis in murine T cell hybridomas and activated peripheral T cells [12]; ricin-induced apoptosis of Madin-Darby canine kidney cells [13]; and tumour necrosis factor-induced apoptosis of a myeloid leukemic cell line [14]. Despite its widespread use in neutrophil studies, its specific effects on the neutrophil biology are not fully understood or studied.

The reported biological effects of DFP on neutrophils are conflicting. Some studies found that DFP has no effect on neutrophil oxidant production, metalloproteinase release, migration [15,16] or phagocytosis [1]. Other studies, however, have reported that DFP decreases the rate of hydrogen peroxide production by neutrophils following stimulation with phorbol myristate acetate (PMA) [17]; suppresses oxygen radical formation from guinea pig neutrophils stimulated with complement-treated zymosan [18]; and suppresses neutrophil phagocytosis [19] and migration [20].

We sought to investigate the effects of DFP on neutrophil functions at a dose (2.5 mM) commonly used in experimental studies [20], specifically focusing on apoptosis and priming of oxidative burst activity. We report differential effects of DFP on neutrophil apoptosis and priming of oxidative burst activity, reflected in suppression of constitutive apoptosis and the priming of oxidative burst function.

## Methods

### Neutrophil Isolation and Culture

We obtained up to 60 mL of whole blood from healthy volunteers, drawing blood into heparinized tubes. We isolated neutrophils by dextran sedimentation and centrifugation through a discontinuous Ficoll gradient as previously described [21]; cell populations were consistently >95% neutrophils, and viability as assessed by trypan blue exclusion routinely exceeded 95%. Neutrophils were resuspended in polypropylene tubes at a concentration of 1 × 10^6 ^cells/mL in supplemented DMEM with 10% fetal bovine serum and 1% penicillin/streptomycin solution (Gibco/BRL).

### Reagents

Antibodies (dilutions; suppliers) used for these studies were murine monoclonal anti-caspase-8 (1:500; Calbiochem), rabbit polyclonal anti-cleaved-caspase-3 (1:500; Calbiochem), murine monoclonal anti-beta-actin (1:4000; Sigma), anti-mouse IgG HRP-conjugated (1:4000; GE Health Care) and anti-rabbit IgG HRP-conjugated (1:4000; GE Health Care).

Diisopropyl fluorophosphate (DFP), a serine protease inhibitor, was purchased from EMD Biosciences. Lipopolysaccharide (LPS) (E. coli Serotype 0111:B4) was purchased from Sigma.

### Neutrophil Elastase Activity Assay

We measured NE activity using a fluorimetric substrate (MeOSuc-Ala-Ala-Pro-Val AMC, Biomol, USA). Briefly, 2 × 10^7 ^cells were lysed in 400 μL of chilled lysis buffer (10 mM Tris, pH 7.4, 150 mM NaCl, 5 mM EDTA, 1% Triton X-100, 10 mM NaF, 1 mM PMSF, 1 mM Na_3_VO_4_, 10 μg/mL leupeptin, 10 μg/mL aprotinin). After measuring protein concentration, 50 μL of cell lysate supernatant was incubated with sample buffer in a 96-well plate at room temperature for 1 hour. The plate was washed four times with sample buffer and 50 μL of a specific substrate for NE was added to the 96-well plate. The plate was then incubated for 4 hours at 37°C. Fluorescence was measured using a fluorimetric plate reader (Fluoroskan) at an excitation wavelength of 355 nm and an emission wavelength 460 nm. Data were analyzed using Ascent Software. NE activity was expressed as arbitrary fluorimetric units (AFU).

### Neutrophil Degranulation Study

We measured neutrophil degranulation by measuring peroxidase release as described [22]. We plated 1.5 × 10^5 ^neutrophils in triplicate in a 96 well tissue culture plate (Sarstedt Microtest Plate). We added 20 μL of control buffer with or without DFP (2.5 mM), then incubated cells at 37°C in a humidified incubator for 1 hour. At the end of the incubation period, the peroxidase reaction was started by adding 70 μL of 2.8 mM TMB in PBS and 60 μL of 1 mM hydrogen peroxide. After 1 minute of incubation at room temperature, the reaction was blocked with 50 μL of stop solution (500 μL of 10 mM sodium azide in 4 N of acetic acid).

Oxidation of TMB was then monitored at 620 nm using a microplate reader (Multiskan Plate Reader, Labsystems). Data were analyzed using Ascent software. The peroxidase activity released in the extracellular environment was expressed as a percentage of the total peroxidase activity of 1.5 × 10^5 ^neutrophils. The total peroxidase activity (100%) was extrapolated from the linear part of calibration curves prepared by assaying the peroxidase activity of different numbers of neutrophils in the presence of 0.02% CTAP (cetyltrimethylammonium bromide).

### Quantification of Constitutive Apoptosis

We measured percentage of neutrophil apoptosis by flow cytometry, quantifying the amount of hypodiploid DNA formation as the uptake of propidium iodide in Triton-X-100 permeabilized cells as previously described [21,23]. After 20 hours of cell culture incubation, Triton-X-100 permeabilized neutrophils were incubated with propidium iodide (50 μg/mL) and analyzed using a BD FACS CANTO Flow Cytometer. Data were analyzed using BD FACS DIVA software. A minimum of 10 000 events were collected and analyzed at excitation wavelength of 488 nm and emission wavelength of 690 nm.

### Western Blot Studies

We lysed 3 × 10^6 ^neutrophils in lysis buffer (10 mM Tris, pH 7.4, 150 mM NaCl, 5 mM EDTA, 1% Triton X-100, 10 mM NaF, 1 mM PMSF, 1 mM Na_3_VO_4_, 10 μg/mL leupeptin, 10 μg/mL aprotinin). Cell lysates were run on a 12% SDS-PAGE gel, transferred to nitrocellulose (Amersham Pharmacia Biotech), and probed with the appropriate primary antibody. Bands were detected with an HRP-conjugated secondary antibody at a dilution of 1:4000 using the ECL Western blotting detection system (Amersham Pharmacia Biotech). Blots were stripped and reprobed with a monoclonal antibody to beta-actin at a 1:4000 dilution, to confirm equal loading of proteins.

### Caspase-3 Activity Assay

We measured caspase-3 activity using a fluorimetric substrate (Ac-DEVD-AMC, Biomol, USA). We lysed 2 × 10^7 ^cells in 400 μL of chilled lysis buffer (10 mM Tris, pH 7.4, 150 mM NaCl, 5 mM EDTA, 1% Triton X-100, 10 mM NaF, 1 mM PMSF, 1 mM Na_3_VO_4_, 10 μg/mL leupeptin, 10 μg/mL aprotinin). After measuring protein concentration, 25 μL of cell lysate supernatant was incubated with 50 μL of a specific substrate (Ac-DEVD-AMC) for caspase-3 in a 96-well plate. Fluorescence was measured using a fluorimetric plate reader (Fluoroskan) at an excitation wavelength of 355 nm and an emission wavelength of 460 nm. Data were analyzed using Ascent Software. Caspase-3 activity was expressed as arbitrary fluorimetric units (AFU).

### Caspase-8 Activity Assay

We measured caspase-8 activity using a colorimetric substrate (IETD-pNA, BioVision CA USA). We lysed 2 × 10^7 ^cells in 400 μL of chilled lysis buffer (10 mM Tris, pH 7.4, 150 mM NaCl, 5 mM EDTA, 1% Triton X-100, 10 mM NaF, 1 mM PMSF, 1 mM Na_3_VU_4_, 10 μg/mL leupeptin, 10 μg/mL aprotinin). After measuring protein concentration, 75 μL of cell lysate supernatant was incubated with 5-7.5 μL of a specific substrate (IETD-pNA) for caspase-8 in a 96-well plate. Plates were incubated at 37°C for 1 hour and color development measured using a colorimetric plate reader (LabSystems Multiskan; Ascent Software) at 405 nm; caspase-8 activity was expressed as absorbance at 405 nm.

### Quantification of Exteriorization of Phosphatidylserine

We measured early events in apoptosis by flow cytometry, quantifying the binding of Annexin V to exteriorized PS [24]. After 5 hours of cell culture, neutrophils were incubated with Annexin V conjugated to the fluorochrome FITC (R&D Systems) and analyzed using a BD FACS CANTO Flow Cytometer with BD FACS DIVA software. A minimum of 10 000 events were collected and analyzed at excitation wavelength of 488 nm and emission wavelength of 518 nm.

### Quantification of Oxidative Burst Activity

We measured oxidative burst activity by flow cytometry, quantifying the conversion of dihydrorhodamine 123 (DHR) to rhodamine 123 as previously described [25]. Neutrophils were incubated with 1 μM of DHR (Invitrogen) at 37°C for 5 minutes followed by incubation with 10^-7 ^M N-Formyl-Met-Leu-Phe (fMLP) for 10 minutes at 37°C. A minimum of 10 000 events were collected and analyzed at excitation wavelength of 488 nm and emission wavelength of 518 nm.

### Quantification of Hydrogen Peroxide Production

We measured hydrogen peroxide production using the Amplex Red Hydrogen Peroxide Kit (Invitrogen) following the manufacturer's instructions. Briefly, after incubating neutrophils with appropriate stimuli, cells were washed, then resuspended in Krebs-Ringer Phosphate (KRPG) buffer. We incubated 2 × 10^4 ^cells with Amplex Red reaction mixture with 10^-7 ^M of fMLP at 37°C for 3 hours. Fluorescence was measured using a fluorimetric plate reader (Fluoroskan) at an excitation wavelength of 544 nm and an emission wavelength of 590 nm. Data were analyzed using Ascent Software. Hydrogen peroxide production was calibrated against a standard curve and was represented in μM.

### Statistical Analysis

Results are reported as the mean ± standard deviation, unless otherwise noted. The paired Student's t test was used to compare continuous variables. The alpha level for statistical significance was set at *p *< 0.05. Analyses were performed using SPSS Statistics 15.0.

## Results

### DFP suppresses neutrophil elastase activity but not neutrophil degranulation

**Figure 1:**
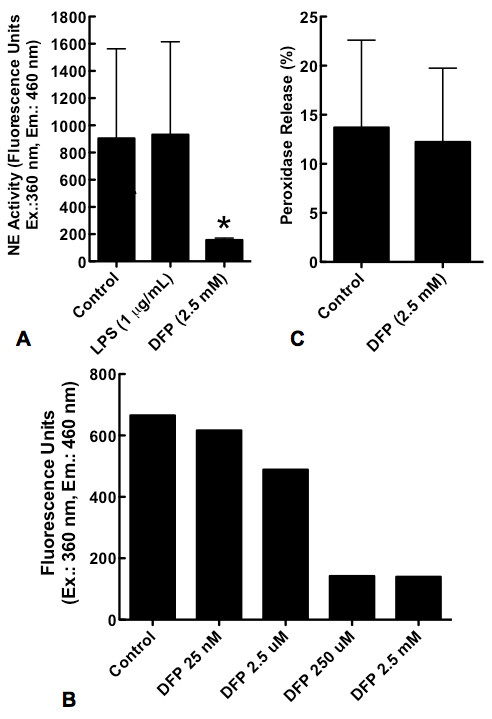
**Effect of DFP on NE activity and neutrophil degranulation**. Human neutrophils were incubated in medium alone (Control), with LPS (1 μg/mL) or with DFP at increasing doses (25 nM, 2.5 μM, 250 μM, and 2.5 mM) for 5 hours. Cells were then lyzed and NE activity was measured at an excitation wavelength of 360 nm and emission wavelength of 460 nm; results are represented as arbitrary fluorescence units. **A**. NE activity of neutrophils treated with or without LPS or DFP (2.5 mM). Data represent the mean ± SD of 9 separate experiments. *P = 0.010. **B**. NE activity of neutrophils treated with increasing doses of DFP; results are from a single experiment. **C**. Human neutrophils were incubated with or without DFP (2.5 mM) for 1 hour and peroxidase release was measured as described in Materials and Methods. Peroxidase release was expressed as percentage of the total peroxidase activity of 150 000 neutrophils treated with 0.02% CTAB. Data represent mean ± SD of 4 separate experiments. *P *= NS.

DFP is an irreversible serine protease inhibitor. We first sought to confirm the effect of DFP on neutrophil endogenous serine protease activity by quantifying neutrophil elastase (NE) activity in neutrophils that had been exposed to DFP (2.5 mM) for 5 hours. DFP significantly inhibited neutrophil NE activity to levels less than 20% of those of control cells (Figure 1A). In contrast, LPS (1 μg/mL) - a stimulus known to inhibit constitutive neutrophil apoptosis [14] had no effect on NE activity (Figure 1A). The inhibition of NE activity by DFP was dose dependent (Figure 1B). NE is stored in neutrophil azurophilic granules. Since reduced NE activity might reflect impaired degranulation, we sought to determine whether DFP suppresses degranulation of neutrophils by measuring peroxidase release in neutrophils that were treated with or without DFP (2.5 mM) for 1 hour. DFP did not inhibit neutrophil degranulation (Figure 1C).

### DFP inhibits neutrophil constitutive apoptosis as measured by hypodiploid DNA formation

**Figure 2:**
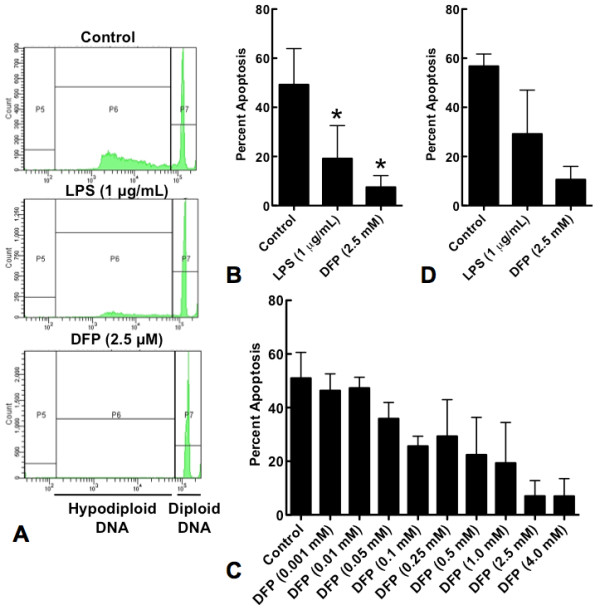
**Effect of DFP on neutrophil constitutive apoptosis**. Human neutrophils were incubated alone (Control), with LPS (1 μg/mL) or with increasing doses of DFP for 20 hours. Cells were permeabilized with Triton X-100 and then stained with propidium iodide (50 μg/mL). **A**. Mean fluorescence values are shown for a minimum of 10 000 cells for each condition and are representative of 3 determinations from 13 separate experiments. **B**. Rate of apoptosis (hypodiploid DNA) of neutrophils treated with or without LPS or DFP (2.5 mM) are represented. Data represent mean ± SD of 15 separate experiments. *P < 0.05. **C**. Rate of apoptosis (hypodiploid DNA) of neutrophils treated with or without increasing doses of DFP. Data represent mean ± SD of 2 to 8 separate experiments. **D**. Rate of apoptosis (hypodiploid DNA) of neutrophils treated with LPS or DFP (2.5 mM) from control cells cultured in serum free medium. Results are mean ± SD of 2 separate experiments.

Quiescent neutrophils are constitutively apoptotic; inflammatory stimuli such as LPS inhibit neutrophil apoptosis. Neutrophils were cultured with or without LPS or DFP for 20 hours and apoptosis quantified as the uptake of propidium iodide. LPS (1 μg/mL) significantly inhibited hypodiploid DNA formation compared to control neutrophils. DFP (2.5 mM) caused significantly greater inhibition (Figure 2A &2B), in a dose-dependent manner (Figure 2C). We confirmed that isopropanol, the vehicle in which DFP is dissolved in, did not inhibit neutrophil hypodiploid DNA formation (data not shown). Similar results were obtained when neutrophils were cultured in serum free media (Figure 2D).

### DFP alters caspase-3 and caspase-8 processing and activity

**Figure 3:**
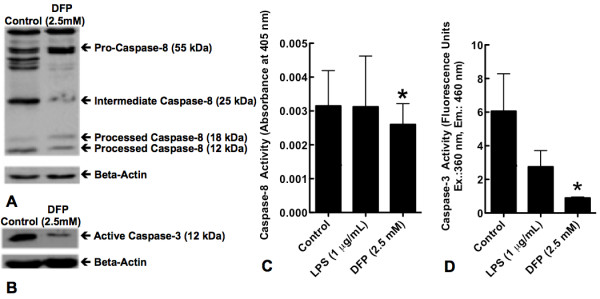
**Effect of DFP on processing of pro-caspases-3 and -8 and caspases-3 and -8 activity**. Human neutrophils were incubated alone (Control) or with DFP 2.5 mM for 5 hours. Cells were then lyzed and lysates were separated on 12% SDS-PAGE gel and specific antibodies were used to evaluate the pattern of caspase-8 (**A**) and caspase-3 (**B**) processing. Blot is representative of 3 separate experiments. **C**. Human neutrophils were incubated alone (Control), with LPS (1 μg/mL) or with DFP (2.5 mM) for 5 hours. Cells were then lyzed. Caspase-8 and caspase-3 activities were measured using specific colorimetric and fluorimetric substrates respectively. Caspase-8 (**C**) activity is represented as absorbance at 405 nm & caspase-3 (**D**) activity is represented as fluorescence units at excitation wavelength of 360 nm and emission wavelength of 460 nm. Data represent mean ± SD of 4 separate experiments. *P = 0.243 for caspase-8; *P = 0.02 for caspase-3.

Caspases are synthesized as pro-enzymes that are cleaved at conserved tetra-or pentapeptide amino acid sequences adjacent to aspartic acid residues to form catalytically active enzymes. Spontaneous neutrophil apoptosis can be initiated via either the extrinsic pathway as a consequence of caspase-8 activation following death receptor engagement [26] or the intrinsic pathway as a consequence of loss of mitochondrial transmembrane potential with activation of caspase-9 [27]. Both pathways result in the activation of the downstream effector, caspase-3. Since caspase activation precedes DNA degradation, we studied the effects of DFP on the processing of caspases-3 and -8 at 5 hours. Consistent with an inhibitory effect on apoptosis, pro-caspase-8 was significantly increased (Figure 3A), while the active 12kDa form of caspase-3 was significantly reduced in DFP treated neutrophils (Figure 3B). Moreover DFP inhibited both caspase-8 activity (Figure 3C), and caspase-3 activity (Figure 3D). The inhibition of caspase-3 activity by DFP was significantly greater than that induced by exposure to LPS (*p *<0.05).

### DFP significantly increases exteriorization of phosphatidylserine in neutrophils

**Figure 4:**
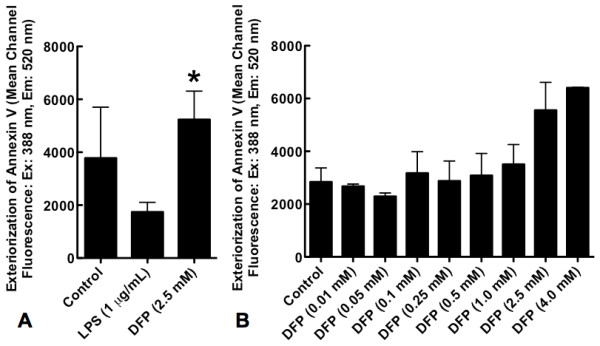
**Effect of DFP on exteriorization of phosphatidylserine quantified as the uptake of Annexin V**. Human neutrophils were incubated alone (Control), with LPS (1 μg/mL) or with increasing doses of DFP for 5 hours. Cells were centrifuged and incubated with FITC-conjugated Annexin. Phosphatidylserine exteriorization was detected as mean channel fluorescence at an excitation wavelength of 388 nm and emission wavelength of 520 nm. **A**. Phosphatidylserine exteriorization of neutrophils treated with or without LPS or DFP (2.5 mM). Data represent mean ± SD of 4 separate experiments. *P = 0.349 versus controls, p < 0.001 versus LPS. **B**. Phosphatidylserine exteriorization of neutrophils treated with or without various doses of DFP. Data represent mean ± SD of 2 to 3 separate experiments.

We next sought to determine the effects of DFP on a separate early event in apoptosis, the exteriorization of phosphatidylserine (PS) on the cell membrane. Contrary to the effects of DFP on hypodiploid DNA formation, culture of neutrophils with DFP (2.5 mM) for 5 hours significantly increased the amount of exteriorized PS as demonstrated by increased binding of Annexin V (Figure 4A). Increased exteriorization of PS was dose-dependent (Figure 4B). In contrast, culture of neutrophils with LPS (1 μg/mL) for 5 hours suppressed the exteriorization of PS (Figure 4A). Thus DFP exerts differential effects on early and late events in the progression of apoptosis. Isopropanol had no effect on the exteriorization of phosphatidylserine (data not shown).

### DFP significantly suppresses neutrophil priming for oxidative burst activity and production of hydrogen peroxide (H_2_O_2_)

**Figure 5:**
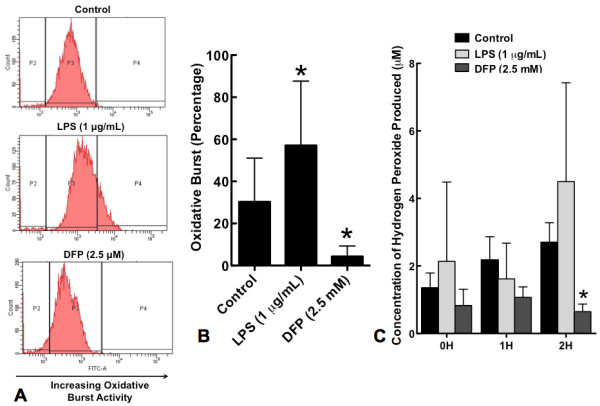
**Effect of DFP on PMN oxidative burst activity and hydrogen peroxide production**. Human neutrophils were incubated alone (Control), with LPS (1 μg/mL) or with DFP (2.5 mM) for 2 hours. Cells were then incubated with 1 μM of DHR followed by incubation with 10^-7 ^M fMLP. Cells were analyzed by flow cytometry to detect the conversion of DHR 123 to rhodamine 123. **A**. Mean fluorescence values are shown for a minimum of 10 000 cells for each condition and are representative of 9 separate experiments. **B**. Oxidative burst activity of neutrophils treated with or without LPS or DFP (2.5 mM). Data represent mean ± SD of 9 separate experiments. *P < 0.002. **C**. Neutrophils were incubated alone (control), with LPS (1 μg/mL) or with DFP (2.5 mM) for 0, 1 and 2 hours. 2 × 10^4 ^cells were incubated with Amplex Red reaction mixture and 10^-7 ^M fMLP. Hydrogen peroxide production was measured using fluorimetric reader, and expressed in μM. Data represent mean ± SD of 7 separate experiments. *P = 0.015

Stimuli such as LPS that delay apoptosis typically prime neutrophils for enhanced oxidative burst activity in response to stimuli such as fMLP [28]. We therefore assessed the effects of DFP on neutrophil oxidative burst activity as measured by the conversion of DHR 123 to rhodamine 123. Neutrophils were incubated with or without LPS (1 μg/mL) or DFP (2.5 mM) for 2 hours. Cells were then incubated with 1 μ M of DHR, with 10^-7 ^M of fMLP. When compared to neutrophils which were cultured alone, LPS-primed neutrophils showed a significant increase in oxidative burst activity (Figure 5A &5B). In contrast, neutrophils that were cultured with DFP demonstrated a significant decrease in oxidative burst activity (Figure 5A &5B). Similar results were obtained when DFP was washed from the cells before DHR was added (data not shown). Isopropanol had no effect on oxidative burst activity (data not shown). Whereas LPS stimulated neutrophil production of hydrogen peroxide, DFP (2.5 mM) significantly inhibited fMLP-induced production of hydrogen peroxide (Figure 5C); isopropanol alone was without effect.

## Discussion

Neutrophils are potent cellular effectors of the early innate response to infection and tissue injury, and their unique biology reflects this critical role. Neutrophils contain significant amounts of proteolytic enzymes (neutrophil elastase, cathepsin G and proteinase 3) stored in azurophilic granules [29]; have the capacity to generate reactive oxygen species [30]; express receptors for Fc component of immunoglobulin and have very short lifespan *in vivo *and *in vitro *as a consequence of the activation of a constitutive apoptotic program following their release from the bone marrow [31]. Because of their intracellular stores of potent proteolytic enzymes, cell culture studies routinely employ inhibitors to prevent artefactual degradation of intracellular proteins. The effects of these inhibitors are poorly characterized.

DFP is an irreversible serine protease inhibitor that permeates intact cells and intracellular granules to prevent proteolysis before cellular barriers are disrupted by homogenization or detergents [1]. It is widely used in experiments that involve neutrophils. Here, we confirmed that DFP neutralizes endogenous protease activity, and specifically that of neutrophil elastase. However we also showed that this inhibition alters key neutrophil functions, including the capacity to undergo spontaneous programmed cell death and to induce an oxidative burst in response to formylated peptides.

DFP significantly suppresses neutrophil constitutive apoptosis, and to a greater extent than LPS - a well-known inhibitor of neutrophil apoptosis. This inhibition is associated with reduced processing of pro-caspases-3 and -8, and suppression of the activity of caspases-3 and -8, resulting in reduced generation of hypodiploid DNA.

Exteriorization of PS serves as recognition ("eat-me") signal for the phagocytosis of apoptotic cells [32]. Exteriorization of PS is thought to occur downstream of caspase activation in some cell types [33] and is enhanced by reactive oxygen species [34]. We found that despite the inhibition of hypodiploid DNA formation, and caspase activity, DFP enhanced the exteriorization of PS in a dose-dependent fashion, suggesting that exteriorization of PS can occur independently of the enzymatic changes of apoptosis. Balasubramanian et al showed that PS exteriorization can occur through a mechanism that is independent of cytochrome *c *release, caspase activation, and DNA fragmentation [35]. DFP may directly influence the activity of flippases and floppases or lipid scramblase, increasing the exteriorization of PS.

We also demonstrated that DFP has a significant inhibitory effect on the priming of neutrophils to respond to fMLP and release reactive oxygen species. This inhibition was evidenced as reduced conversion of DHR 123 to rhodamine 123 and reduced release of hydrogen peroxide. These results raise the possibility that serine proteases, in addition to their direct role in the intracellular killing of microorganisms, may also participate in indirect killing by enhancing the neutrophils' ability to respond to stimuli, such as the bacterial tripeptide fMLP with an increased generation of reactive oxygen species. Of note, DFP did not suppress oxidative burst significantly in the absence of fMLP.

The demonstration of the modulation of events in the evolution of apoptosis by DFP underscores the potential roles of serine proteases in the regulation of apoptosis. Our results further suggest that neutrophil serine proteases enhance the priming of neutrophils for oxidative burst activity.

## Conclusion

On the one hand, our results suggest additional roles for serine proteases in the orchestration of an innate immune response through their effects in enhancing neutrophil priming for oxidative burst and apoptosis. On the other hand, they underscore a potential drawback in using DFP in neutrophil studies to prevent proteolysis and to perform granulocyte kinetic studies *in vivo*, and suggest that caution must be taken in interpreting the results of studies in which DFP has been used for its anti-protease activity.

## Competing interests

The authors declare that they have no competing interests.

## Authors' contributions

JLYT designed and planned all experiments, performed most of the experiments, analyzed and interpreted all the data, prepared and revised the manuscript. JCP performed some of the experiments. JCM obtained funding, participated in analysis and interpretation of the data, and revised the manuscript. All the authors have read and approved the final manuscript.
